# Sex Hormones and Optic Nerve Disorders: A Review

**DOI:** 10.3389/fnins.2019.00057

**Published:** 2019-02-11

**Authors:** Raffaele Nuzzi, Simona Scalabrin, Alice Becco, Giancarlo Panzica

**Affiliations:** ^1^Eye Clinic, Department of Surgical Sciences, AOU Città della Salute e della Scienza, Ophtalmic Clinic, University of Turin, Turin, Italy; ^2^Laboratory of Neuroendocrinology, Department of Neuroscience Rita Levi-Montalcini, University of Turin, Turin, Italy; ^3^Neuroscience Institute Cavalieri-Ottolenghi, Orbassano, Italy

**Keywords:** gonadal hormones, estrogens, hormone therapy, eye disorders, glaucoma, optic nerve diseases, sex-related differences

## Abstract

**Aim:** This review article presents a comprehensive overview of the literature on sex hormones (estrogens, androgens, progesterone) and optic nerve disorders, with a discussion of the implications for therapy and prevention.

**Methods:** Epidemiological, pre-clinical and clinical studies were reviewed.

**Results:** Analysis of the biological basis for a relationship between eye diseases and sex hormones showed that some types of hormones can exert a protective effect either directly on the retina and optic nerve or indirectly by modulating ocular blood flow. For example, it seems that estrogen exposure has a protective effect against glaucoma, whereas its deficit may lead to early onset of the disease. If further studies confirm the data in the literature, estrogen therapy, because of its antioxidant action, may be effective in the treatment of Leber's hereditary optic neuropathy, whereas, in the light of current studies, there does not seem to be an influence of estrogen on non-arteritic anterior ischemic optic neuritis (NAION).

**Conclusions:** Although there is some evidence that in some optic nerve pathologies the sex hormones seem to play an important role there are still too few studies providing evidence for its wider use in clinical practice.

## Introduction

There is growing evidence for interaction between gonadal hormones and diseases involving body systems other than the reproductive organs (Rosner et al., 2013; Eisner, 2015). Some correlations, like those between estrogens and bone metabolism, are well established (Clarke and Khosla, 2010; Eisner, 2015), whereas others, particularly as regards the eye, are less evident. Estrogens, however, seem to exert a neuroprotective effect (Azcoitia et al., 2011; Melcangi et al., 2011), a vascular effect (Toker et al., 2003a; Faria et al., 2011; Schmidl et al., 2015), an intraocular pressure (IOP) regulation effect (Weinreb and Khaw, 2004; Tehrani, 2015) and a support action against lamina cribrosa. These evidences support the hypothesis that sex hormones may be involved in the pathogenesis of eye diseases, such neuropathies as glaucoma, but also retinic diseases, as age-related macular degeneration (AMD), and diseases of the lens, as cataracts. It may also be speculated that hormone replacement therapy (HRT) in postmenopausal women can influence the course of these disorders and be useful in the treatment of eye diseases (Hutchinson et al., 2014; Nuzzi et al., 2018; Table 1).

**Table 1:** Basis of interaction between sex hormones and the eye.

Biological basis	Presence of androgen, estrogen, and progesterone receptors (or the expression of their mRNA) in many different ocular tissues (Rocha et al., 2000; Wickham et al., 2000; Gupta et al., 2005)
	Retina produces neuroactive steroids by local synthesis of cholesterol via the neurosteroidogenic pathway (Cascio et al., 2015)
	Differences in retinal function of human men and women are significant before the age of 50, while at a later age the results are overlapping (Ozawa et al., 2014).
	Women who had undergone hysterectomy during reproductive age have a retinal function worse than other women and men (Ozawa et al., 2014).
	Normal hormonal fluctuations during the menstrual cycle affect the optic nerve in women with severe type 2 diabetes (Naderan, 2018)
	Changes in hormone levels during pregnancy determine physiological changes on most ocular structures (Sanke, 1984; Bolanca et al., 2008)
Epidemiological basis	In women, estrogens exert a protective effect against cataracts until the onset of menopause, when the risk of developing cataracts increases over that for men (Zetterberg and Celojevic, 2015).
	Women are more often affected by closed angle glaucoma and certain forms of AMD (e.g., neovascular AMD), whereas primary open-angle glaucoma and diabetic retinopathy are more prevalent among men (Zetterberg, 2016)
Neuroprotective effect	17β-estradiol and nonfeminizing estrogen analogs protect retinal photoreceptor neurons from glutamate-induced damage (Gotovac et al., 2013).
	Neuroprotective action was found not only for estrogens but also for molecules with similar phenolic components (Sharma et al., 2006).
	Substitutive phytoestrogen therapy has neuroprotective effects similar to those observed using estrogens (Beneyto and Pérez, 2006).
	There is an inferior decrease in retinal ganglion cell density in non-ovariectomized mice and a protective effect against axotomy-induced RGC damage in the ovariectomized mice administered intravitreal exogenous estrogens (Klein et al., 1990).
	Estrogens, via their anti-inflammatory action, inhibit or downregulate cytokine production (Yust-Katz et al., 2014)
Effect on ocular hemodynamics	In central retinal artery and in ophthalmic artery vascular resistance is lower and blood flow velocity is higher in premenopausal women than postmenopausal women and men (Toker et al., 2003a; Faria et al., 2011; Schmidl et al., 2015).
	Infero-temporal retinal artery flow was greater in postmenopausal women receiving HRT than in those not receiving it (Akar et al., 2005b)

*AMD, age-related macular degeneration; RGC, retinal ganglion cell; HRT, hormone replacement therapy*.

This review article presents an overview of the literature on sex hormones and optic nerve disorders, with a discussion of the implications for therapy and prevention.

## Biological and Epidemiological Basis for the Interaction between Sex Hormones and the Eye

As recently reviewed by us in a review aimed at outlining the current knowledge on the possible activity carried out by sex hormones on retinal physiopathology (Nuzzi et al., 2018), the long-held notion that the eye was “sex-neutral” has no significance today, and a number of studies have, on the contrary, provided several data in favor of a close interaction between gonadal hormones and eye function. In brief, androgen, estrogen, and progesterone receptors (or the expression of their mRNA) have been demonstrated in many different ocular tissues, including the lachrymal and meibomian glands, the bulbar palpebral conjunctiva, the corneal surface, the ciliary body of the lens, and the chorioretinal complex (Rocha et al., 2000; Wickham et al., 2000; Gupta et al., 2005). In addition, the retina produces neuroactive steroids by local synthesis of cholesterol via the neurosteroidogenic pathway (Cascio et al., 2015; Nuzzi et al., 2018). Changes in the availability of neuroactive steroid following local insults have been implicated in the adaptation of retinal cells to insults (Guarnieri et al., 2003).

Age and sex may influence the distribution of gonadal hormone receptors throughout ocular tissues, and this has been related with the differences in the epidemiology of certain disorders (Gupta et al., 2005). For example estrogen receptor alpha (ER-α) is present in the retina and retinal pigment epithelium of young women but not in that of postmenopausal women or men (Ogueta et al., 1999), while Munaut et al. (2001) found ER-β in the human retina of male and female subjects. Studies, using multifocal electroretinography, investigated the differences in humans, taking into account not only the gender, but also the age of the subjects; this allowed to demonstrate that the differences in retinal function of men and women were significant before the age of 50, while at a later age the results were overlapping. Comparing the results of all these groups with those of women who had undergone hysterectomy and ovariectomy during reproductive age and so experienced iatrogenic menopause, it emerged that the latter had the worst retinal function, underlining the benefits of exposure to estrogenic cycles (Ozawa et al., 2014).

Such anatomical and physiological differences are also reflected in disease processes. For example, estrogens exert a protective effect against cataracts during women's reproductive years until the onset of menopause, when the risk of developing cataracts increases over that for men (Zetterberg and Celojevic, 2015). Although an age/sex correlation for the incidence of certain diseases has not been well established, some studies have found that women are more often affected by closed angle glaucoma and certain forms of AMD (e.g., neovascular AMD), whereas primary open-angle glaucoma (POAG) and diabetic retinopathy are more prevalent among men (Zetterberg, 2016).

Sex hormones' influence seems to be present not only in pathologies that primarily affect the optic nerve, but also in those in which the involvement of the nerve is secondary, as diabetic retinopathy.

Akar et al. (2005a) examined the effect of the menstrual cycle on the optic nerve head in women with type 2 diabetes and found significant changes (in particular an increase in the neuroretinal rim area and a significant reduction in cup-shape measure) that occur in the luteal phase in women with severe diabetic retinopathy, but that not occur in women with mild diabetic retinopathy and in healthy subjects. The study suggested that normal hormonal fluctuations during the menstrual cycle may affect the optic nerve only in cases of advanced disease.

It has been established that hormones, in particular estrogens and progesterone, cause physiological changes on most ocular structures; these changes can determine, in predisposed subjects, the appearance of new conditions or the evolution of pre-existing pathologies. Naderan (2018) investigated the role that hormonal changes related to pregnancy have on ocular physiopathology; they observed that some new conditions that often occur during pregnancy are ocular dryness, associated with or without contact lens intolerance, chloasma (Bolanca et al., 2008) (a skin pigmentation localized specially in the face and that mostly appears during pregnancy) and ocular ptosis (Sanke, 1984) (the lowering of the eyelids), phenomena which resolve spontaneously in most cases after childbirth. In some ocular structures, during pregnancy, physiological changes occur due to the increased water retention favored by the increase of estrogenic levels. This happens for example in the cornea, causing an increase in the thickness of the central portion, which is expressed with refractive changes (Sharma et al., 2006; Gotovac et al., 2013; Mehdizadehkashi et al., 2014), and at the lens level (Beneyto and Pérez, 2006), with possible appearance of cataract or worsening of a pre-existing condition. Among the pathological ocular conditions most frequently associated with pregnancy there are diabetic retinopathy (Klein et al., 1990), which often progresses rapidly during this period, and central serous chorioretinitis (Liu et al., 2016), of which pregnancy is a risk factor, which resolves spontaneously, usually within 3 months of giving birth. According to some studies (Yust-Katz et al., 2014) also gliomas would show a greater risk of progression during pregnancy, especially if Grade II and III. van Westrhenen et al. (2018) shown that pregnancy can be related both to a clinical deterioration and to an increase in tumor size when imaged with an magnetic resonance imaging scanner. Instead, other pre-existing diseases improve during pregnancy, a phenomenon that supports the neuroprotective effect of sex hormones. Among them are uveitis (Chiam and Lim, 2014), in which both hormonal and immunological changes related to pregnancy play a role, and especially glaucoma, which is positively affected by the action of progesterone, which seems to play a fundamental role, even if not yet fully clarified, in lowering the IOP (Akar et al., 2005b).

To further understand if sex hormones play a role in the pathophysiology of the retina and optic nerve, some studies focused on the analysis of the thickness of the macular region and the retinal nerve fiber layers (RNFL) in patients with polycystic ovary syndrome (PCOS), an endocrine-gynecological pathology characterized by the presence of ovarian microcysts, menstrual irregularities and hyperandrogenism, often associated with alterations of glucose metabolism, in particular with insulin resistance, glucose intolerance and type 2 diabetes. Demir et al. (2013) highlighted, by measuring the RNFL and ganglion cell complex thicknesses by optical coherence tomography (OCT), that the average thickness of the RNLF in the different sectors was significantly higher in women with PCOS than in healthy women. A few years later, the results obtained by this study were extended by another group of researchers (de Souza-Júnior et al., 2015), which showed that PCOS seems to play, thanks to the trophic action of testosterone, a protective role on the RNFL around the optic nerve, increasing its thickness, but only if there are no other metabolic abnormalities, because if these are present, PCOS would seem to perform a negative action, reducing RNFL and total macular thickness. The effect of PCOS at the ocular level also manifests itself in the alterations induced on the ocular hemodynamics: in patients with PCOS ocular blood flow velocity is increased and at the level of the ophthalmic artery the vascular resistance seems to decrease (Örnek et al., 2015).

In light of this evidence, it is clear that sex hormones are involved in the physiological homeostasis of ocular tissues, as evidenced by the presence of specific receptors in various structures that compose the eye, and that in some pathologies these hormones play a role of modulation, protective or pathogenic, according to the specific condition analyzed.

## Neuroprotective Effect of Gonadal Hormones

One of the aims of studies on the interaction between sex hormones and the eye is to evaluate the neuroprotective action these hormones, estrogens in particular, appear to have. Estrogens protect retinal photoreceptor neurons from glutamate-induced damage (Nixon and Simpkins, 2012) and this action is probably mediated by membrane estrogen receptor (GPR30), which is also expressed in the retina (Mangiamele et al., 2017).

Estrogens, but also similar non estrogenic phenolic components, may also exert antioxidant neuroprotective effects (Moosmann and Behl, 1999). This observation suggests the possibility of treatments based with nonfeminizing estrogen analogs and phenolic molecules that, despite having similar neuroprotective effects to estrogen, should show fewer side effects. For example, by studying the ocular function of postmenopausal women using short wavelength perimetry, it has been observed that a substitutive phytoestrogen therapy has effects similar to those observed using estrogens (Eisner and Demirel, 2011).

To determine whether endogenous and exogenous estrogens differed in action, Nakazawa et al. (2006) used a rodent model of axotomy-induced retinal ganglion cell (RGC) degeneration in glaucoma in ovariectomized and non-ovariectomized mice. They observed a significantly reduced decrease in retinal ganglion cell density in the non-ovariectomized mice, suggesting an effect of endogenous estrogens. In addition, they noted a protective effect against axotomy-induced RGC damage in the ovariectomized mice administered intravitreal exogenous estrogens. These results seem to indicate that exogenous hormones have a protective action similar to that of endogenous hormones.

Another possible source of damage to the optic nerve is thought to be the activation of inflammatory pathways by proinflammatory mediators, and by cytokines in particular (Clzonkowska et al., 2006). It has been demonstrated that estrogens, via their anti-inflammatory action, inhibit or downregulate cytokine production (Baker et al., 2004). This beneficial effect may partly explain sex-related differences in certain diseases, and in those with an inflammatory component in particular. The initial phase of many neurodegenerative diseases is characterized by damage caused by a primary event to which the body mounts an inflammatory response. Though activated as a protective response to injury, it also results in further tissue damage. Estrogens could provide protection against such diseases by mitigating the initial inflammatory response associated with them.

Building on the observation that estrogens exert a neuroprotective effect on the retina and optic nerve, researchers applied this knowledge to determine whether other sex hormones such as progesterone had a similar effect. Experimental studies on animal models found no significant differences (Kaldi and Berta, 2004), however, more recent studies have reopened the question, demonstrating that norgestrel, an analog of progesterone, would play a neuroprotective role in retinal degeneration in retinitis pigmentosa (Ruiz Lopez et al., 2017).

## Effect of Gonadal Hormones on Ocular Hemodynamics

An essential action of gonadal hormones is to regulate ocular blood flow (Toker et al., 2003a; Schmidl et al., 2015), which indirectly affects neurotrophism and eye function. Numerous studies have investigated how and to what extent these hormones are involved in blood flow regulation and are implicated in pathogenic processes triggered by hemodynamic abnormalities.

The beneficial effect of estrogens has been correlated with their vasodilatory properties, which, by determining lower vascular resistances, guarantee a greater blood flow, especially in larger vessels (Dechênes et al., 2010; Schmidl et al., 2015). In fact, in central retinal artery and in ophthalmic artery vascular resistance is lower and blood flow velocity is higher in premenopausal women in which endogenous estrogen levels are naturally higher, or in women who received exogenous estrogens (Schmidl et al., 2015), than in postmenopausal women exposed or not to HRT (Toker et al., 2003a; Faria et al., 2011). In contrast, androgens (Dechênes et al., 2010) and progesterone (Viana et al., 2011; Souza et al., 2013) reduce retinal blood flow by antagonizing estrogen activity.

## Sex-Related Differences in Relation to Optic Nerve Disease

There is growing evidence for links between sex hormones and some important optic nerve diseases, including glaucoma, Leber's hereditary optic neuropathy, nonarteritic anterior ischemic optic neuropathy, optic neuritis and optic nerve tumors, especially gliomas. In this section of the review we proceed to collect and summarize the main information currently present in the literature about the association between the aforementioned pathologies and sexual hormones.

### Glaucoma

Glaucoma is a neurodegenerative disease that affects the optic nerve, characterized by the gradual damage of retinal ganglion cells and their axons, that compose the optic nerve (Schmidt et al., 2008).

Since these cells play a critical role in the process of vision, the disease manifests itself with a decrease in visual acuity, of variable entity depending on the type of glaucoma and the modalities of onset (Yücel et al., 2003). It represents the second most common cause of blindness in the world (Tham et al., 2014) and it is therefore important to study in depth all the factors that influence this pathology, including estrogens.

The analysis of various studies (Dewundara et al., 2016) showed that the factors modulating the duration of exposure to estrogens seem to influence the risk of developing a POAG: prolonged exposure to estrogens, such as that occurring in women entering menopause after age 54 (Pasquale and Kang, 2011), leads to a lower risk of developing POAG. On the contrary, all the conditions reducing the duration of exposure to estrogen such as the age at menarche over 13 years (Lee et al., 2003), bilateral ovariectomy before age of 43 (Vajaranant et al., 2014), or spontaneous menopause before age of 45 (Hulsman et al., 2001), but also oral contraceptive therapy (OC) for more than 5 years (Pasquale and Kang, 2011), increase the risk of developing POAG. Finally, during pregnancy, especially during the third trimester, in which estrogen levels are very high, a reduction in IOP is observed (Qureshi, 1995; Qureshi et al., 1996; Naderan, 2018). The role of HRT for menopause is still uncertain (Sator et al., 1997; Toker et al., 2003b; Altinta et al., 2004), although some studies have shown a beneficial effect in terms of reducing the risk of developing POAG (Tehrani, 2015). Another recent study (Shin et al., 2018), conducted on the Korean population, identified a statistically significant association between the risk of developing POAG and female reproductive factors only in the case of early menopause (before age 45); in this study, other factors such as the age of menarche, pregnancy, breastfeeding, use of contraceptive or replacement therapies were not significant in modifying the incidence of POAG.

Several more recent studies confirmed and expanded the hypothesis of the influence of estrogen on glaucoma: it has been highlighted, for example, that estrogens appear involved in the process of formation and drainage of the aqueous humor, key elements in the pathogenesis of glaucoma (Ogueta et al., 1999; Altinta et al., 2004). IOP, which is fundamental for optic nerve tropism, also seems to be influenced by changes in estrogen concentration (Weinreb and Khaw, 2004). Many studies have focused on the estrogen-IOP relationship: (Tehrani, 2015) has produced a review in which, after having verified the existence of epidemiological differences concerning the incidence of glaucoma in the two genders, analyzed not only the action of endogenous estrogens, but also the role of exogenous estrogens, administered as OC therapy or as HRT in menopausal women. From this evaluation it emerges that the IOP and the risk of developing glaucoma vary depending on the hormone concentration: in particular, HRT in postmenopause women would seem to reduce the risk of glaucoma, while in premenopausal women an OC therapy would lead to an increased risk. Tehrani hypothesizes that the opposite effect that the two types of therapy exert on the risk of developing glaucoma may depend both on the different hormone formulation of the two therapies, and on the fact that the OC therapy suppress ovulation, going to alter the physiological hormone levels of the menstrual cycle, which causes a reduction in the total endogenous estrogens production.

Chen et al. (2018) studied the role of estrogen on the regulation of IOP and, consequently, on the status of retinal ganglion cells, evaluating the effect of the lack of aromatase, an enzyme necessary for the production of estrogens. To perform this study both wild type and aromatase knockout mice were compared (based on age and sex). The knockout females showed significantly higher IOP levels compared to wild type females of the same age and significantly lower RGC levels. The male mice, on the other hand, did not show any significant difference between the two groups as regards the IOP, although the 12 week knockouts showed a reduced RGC count compared to the wildtype of the same age. These results further confirm the role of estrogens in regulating IOP, reducing it and ensuring optimal RGC status.

Vajaranant and Pasquae (2012) noted that estrogen may also be involved in the aging process of the optic nerve, which appears to be accelerated in the presence of an estrogenic deficiency. In addition, studies aimed at assessing the possibility of estrogen influencing the topography of the optic nerve head have shown that the menstrual cycle-related fluctuations significantly modify the central area and the edge of the optic nerve head, results that should be taken into consideration in the follow-up of young women with glaucoma (Akar et al., 2004). All these elements would allow us to hypothesize that a reduction in estrogen concentrations leads to a greater susceptibility to the development of glaucoma.

Some studies have focused their attention on genetic analysis, trying to assess the existence of a possible correlation between single nucleotide polymorphisms (SNPs) of genes involved in estrogen metabolism and the development of POAG. For example, SNPs in the endothelial nitric oxide synthase (eNOS3), a gene regulated by estrogens, seem to be effectively correlated with the development of open- angle glaucoma (Ball and Knuppen, 1980; Furchgott and Zawaszki, 1980; Pasquale et al., 2013). Pasquale et al. (2013) have shown that, among women, some SNPs were related to global POAG and to high-pressure POAG, but not to those with low intraocular pressure; in men, however, no type of correlation was observed. Also within the female sample it was observed that the gene most associated with both forms of open angle glaucoma appeared to be that of catechol-O-methyltransferase, an enzyme that is involved in the methylation of two estradiol derivatives, a process that seems to reduce the bioavailability of estrogens. In conclusion, links between the SNPs of the estrogenic pathways and the onset of glaucoma were observed, but only for some types and exclusively in women.

### Leber's Hereditary Optic Neuropathy

Leber's hereditary optic neuropathy (LHON), the most common mitochondrial disease, is due to punctiform mutations of mitochondrial DNA, transmitted by maternal inheritance. LHON is characterized by RGC degeneration, with optic nerve atrophy and loss of central vision (Man et al., 2003; Carelli et al., 2004). It affects males more often than females and has its onset in young adulthood. Since it is a genetic disease with maternal inheritance, its incidence should theoretically be equal between the sexes. Its higher occurrence in males could be due to the protective effect of estrogens in women who, though carrying the genetic mutation, do not develop the disease.

To test this hypothesis, Giordano et al. (2011) used as a model cybrids, i.e., cytoplasmic hybrids or eukaryotic cell lines produced by the fusion of a whole cell (cytoplasm and nucleus) within a cytoplast (an enucleated cell), which were treated with ICI 182780, an estrogen receptor antagonist, during the first stage of the study. The cybrids presenting the typical mutations of LHON presented higher oxygen reactive species levels than the control cybrids, with greater apoptosis and less cell viability. In the second stage of the study, 17β-estradiol was administered to both groups of cybrids, which led to an improvement in the experimental group due to the activation of superoxide dismutase by E_2_ and activation of mitochondrial biogenesis and a slight, albeit significant, increase in energy production. These results appear to corroborate the potential role of estrogens in preventing the development of LHON in females. The findings also provide an explanation for a hormone-correlated metabolic basis for the prevalence of LHON in males, and open horizons for designing treatments with estrogens or their analogs.

Building on these findings, the same research group conducted a study (Pisano et al., 2015) again using cybrids and observed that estrogen administration protected the mutated cells via the activation of ERβ in the mitochondria of the RGCs. The protective effect of estrogen was confirmed by the fact that administration of receptor antagonist inhibited the protective action of the estrogens. The aim of the study was to determine whether use of ERβ as a target for potential estrogen therapy could prevent or delay LHON in carriers of the genetic mutations associated with its development: findings provide evidence that phytoestrogen can correct *in vitro* the cellular pathologic phenotype associated with LHON mutations, in both cybrids and patient derived fibroblasts. If further studies confirm these initial findings, estrogen therapy may have significant implications for the course of the disease, since no current therapy has a particularly positive effect in the acute phase.

### Nonarteritic Anterior Ischemic Optic Neuropathy

NAION refers to optic nerve stroke caused by ischemia of the axons of the RGCs (Hayreh, 1974). It is the most common cause of sudden loss of vision associated with the optic nerve after age 50 years (Miller, 1980).

Studies using rodent models have evaluated whether estrogens have a protective action (Bernstein et al., 2007). NAION was induced in ovariectomized mice given either estradiol or placebo. The results showed that although estrogen-correlated transcript expression was augmented, it was not influenced by systemic estrogen administration and there was no difference in the number of surviving RGCs, indicating that probably estrogens exert no protective action against NAION.

### Optic Neuritis

Optic neuritis is an inflammation of the optic nerve often associated with multiple sclerosis (MS): it manifests as the first sign of disease in 20% of cases (Miller et al., 2005) and affects 30–70% of persons with MS during their lifetime (Frohman et al., 2008).

Studies investigating sex-related differences in RNFL thinning measured using OCT at 6 months after the acute event (Costello et al., 2012) showed that, on average, men were older than women, were more likely to have relapsing or remitting MS, and less thinning of the RNFL, with greater changes as compared to basal values.

Taking as an example the demyelinating optical neuritis, characteristic of MS, it is important to underline that, although in some aspects the pathogenesis of optic nerve and retinal pathologies present common elements, there is an important difference due to the fact that retinal pathologies may be due to the injury or malfunction of various cell types, including photoreceptors, bipolar cells, ganglion cells and glia cells. Optic nerve neuropathies depend almost exclusively on damage to ganglion cells or nerve fibers; the latter include pathologies affecting the vessels, responsible for vascularization and trophism of the nerve, and those affecting myelin, which covers the axons constituting the nerve.

Various studies analyzed the possible relationship between sex hormones and myelin formation, especially in the context of demyelinating diseases such as MS: Sicotte et al. (2002), starting from the observation that women with MS in pregnancy have a reduction of relapses, showed that administration of estriol, a hormone typical of pregnancy, showed a beneficial effect on the history of MS even in non-pregnant women; Soldan et al. (2003), subsequently identified as the likely cause of this phenomenon the immunomodulatory effect of oral estriol therapy. More recently, scientists tried to understand the effects of a combination therapy of 17-β estradiol and intravenous adipose-derived mesenchymal stem cell (ADSC) transplantation. Results showed that 17-β estradiol increased the efficacy of intravenous ADSCs in remyelination of corpus callosum axons (Ragerdi Kashani et al., 2012).

Kim et al. (2018) tried to understand through which mechanism estrogens favor the remyelination and they have discovered that pathways involved are that of the ER β. Further research has shown that the activation of this receptor occurs both at the level of the brain immune cells (CD11c^+^), and on the oligodendrocytes; the action on CD11c^+^ immunomodulatory cells would seem essential to favor the correct maturation of oligodendrocytes, suggesting the need for a synergistic action of estrogen on the two categories of cells.

Progesterone and its derivatives have also been shown to have beneficial activities in this field, being able not only to reduce the extent of myelin sheath loss in MS, but also to promote the formation of new myelin (El-Etr et al., 2015).

The possible role of androgens has also been examined because, since these diseases affect males less frequently than women. A protective effect of testosterone has been hypothesized; some studies have shown that testosterone, acting on neural androgen receptors, stimulates myelin repair and has an anti-inflammatory action (Hussain et al., 2013).

Therefore, sex hormones seem to play a role in myelin regeneration during demyelinating pathologies, evidence that could play an important role not only in the specific therapy of these pathologies, but also in associated manifestations that present the same pathogenesis, such as optic neuritis.

Another inflammatory disease of the optic nerve in which a relationship between hormones and neuroretinal disease has been suggested is neuromyelitis optica spectrum disorder with aquaporin-4-immunoglobulin G (NMOSD-AQP4) (Wingerchuk et al., 2015), a relapsing demyelinating inflammatory disease characterized by uni- or bilateral optic neuritis, acute myelitis, and higher prevalence among females (Wingerchuk, 2009).

In their study on the relationship between sex and clinical manifestations of the disease, Kim et al. (2017) found that its onset is later in men than in women (average 49 vs. 41 years of age), neuritic attacks are less frequent at onset (17 vs. 44%), and are less frequent during the course of the disease (0.08 vs. 0.27 per year) in men than in women. Males more often tend to develop an isolated form of myelitis (67 vs. 28%). Visual evoked potential testing showed a shorter latency of P100 in men. Men were also noted to have fewer acute optic neuritis attacks, independent of age at onset of the disease. However, this type of optic neuritis is less known and deserves further study.

### Optic Nerve Tumors: Glioma and Meningioma

Some researchers investigated the role of sex hormones in the pathogenesis of various tumors, including the ones that frequently affect the optic nerve, gliomas and meningiomas.

Different studies evaluated the existence of correlation between reproductive factors and the risk of the onset of glioma. Although most of the reproductive factors (eg., age at menopause, number of pregnancies) were not related to the development of this type of cancer (Felini et al., 2009), an increased age at menarche was found to be a potential risk factor for glioma development according to various studies (Felini et al., 2009; Qi et al., 2013; Anic et al., 2014; Krishnamachari et al., 2014). Specifically, Anic et al. (2014) found that this increased susceptibility to the development of glioma in women with an older age at menarche is present only in premenopausal women, whereas this correlation is lacking after menopause. The analysis by Felini et al. (2009) found that the correlation glioma- age at menarche is strong only in cases of non-glioblastoma histologies. These findings would agree with the hypothesis that prolonged exposure to estrogen plays a protective role, reducing the risk of developing this tumor, a hypothesis partly supported by data from the analysis of the relationship between glioma and OC and/or HRT, although in this case results are at times conflicting. Some studies in literature agree in identifying a reduction of the risk of glioma in subjects undergoing hormone therapy, be it OC (Qi et al., 2013; Anic et al., 2014; Krishnamachari et al., 2014) or HRT (Qi et al., 2013; Krishnamachari et al., 2014), others, on the contrary, identified an increase in risk for HRT based on estrogen only, but not for HRT based on estroprogestestinal (Andersen et al., 2013a; Benson et al., 2015).

Other researchers focused attention on another frequent tumor that affects the optic nerve, the meningioma. In this case the risk of developing tumor is higher in women who had undergone OC (Michaud et al., 2010; Claus et al., 2013) or HRT (Michaud et al., 2010; Andersen et al., 2013b; Benson et al., 2015); the type of HRT most likely associated with meningioma is not yet clear, since according to some studies the risk is higher with therapies based only on estrogens (Benson et al., 2015), while according to others it is higher in therapies with combination of estroprogestestinal (Andersen et al., 2013b).

Although sex hormones would seem to be related to the onset of these tumors, apparently in opposite way in the case of glioma and meningioma, studies in this area are too few to be able to define these results as definitive.

## Conclusions

This review presents the current state of the art of the possible relationship between sex hormones and optic nerve disorders (Table 2). As noted in other ophthalmic disorders, as retinal diseases (Nuzzi et al., 2018), sex hormones seem to be involved in optic nerve disorders and may play an important role in their prevention and in their therapeutic use. Although current evidence is still insufficient for drawing conclusions, it does provide starting points for experimental and clinical studies that may ultimately inform clinical practice.

**Table 2:** Role of sex hormones in optic nerve disorders. For and against evidences, with type of correlation.

**Protective effect (+)**	**No difference or non-protective effect (–)**	**Unclear effect (±)**
**Disease**	**Estrogen**	**Studies**
Glaucoma	+	Dewundara et al., 2016
	+	Vajaranant and Pasquae, 2012
	+	Akar et al., 2004
	+	Shin et al., 2018
	+	Chen et al., 2018
LHON	+	Giordano et al., 2011
	+	Pisano et al., 2015
NAION	–	Bernstein et al., 2007
Optic Neuritis	±	Costello et al., 2012
Glioma and meningioma	±	Anic et al., 2014
	±	Felini et al., 2009
	±	Benson et al., 2015
	+	Krishnamachari et al., 2014
	+	Qi et al., 2013.
	–	Andersen et al., 2013a
	–	Michaud et al., 2010
	–	Claus et al., 2013

*LHON, Leber's hereditary optic neuropathy; NAOIN, non-arteritic anterior ischemic optic neuropathy*.

## Author Contributions

All authors listed have made a substantial, direct and intellectual contribution to the work, and approved it for publication.

### Conflict of Interest Statement

The authors declare that the research was conducted in the absence of any commercial or financial relationships that could be construed as a potential conflict of interest.

## Abbreviations

ADSC: adipose-derived mesenchymal stem cell
AMD: age-related macular degeneration
ER-α: estrogen receptor alpha
HRT: hormone replacement therapy
IOP: intraocular pressure
LHON: Leber's hereditary optic neuropathy
MS: multiple sclerosis
NAION: nonarteritic anterior ischemic optic neuropathy
OC: oral contraceptive therapy
OCT: optical coherence tomography
PCOS: polycystic ovary syndrome
POAG: primary open-angle glaucoma
RGC: retinal ganglion cell
RNFL: nerve fiber layer
SNPs: single nucleotide polymorphisms.
